# MR constrained simultaneous reconstruction of activity and attenuation maps in brain TOF-PET/MR imaging

**DOI:** 10.1186/2197-7364-1-S1-A55

**Published:** 2014-07-29

**Authors:** Abolfazl Mehranian, Habib Zaidi

**Affiliations:** Division of Nuclear Medicine and Molecular Imaging, Geneva University Hospital, CH-1211 Geneva, Switzerland

The maximum likelihood estimation of attenuation and activity (MLAA) algorithm has been proposed to jointly estimate activity and attenuation from emission data only. Salomon *et al* employed the MLAA to estimate activity and attenuation from time-of-flight PET data with spatial MR prior information on attenuation. Recently, we proposed a novel algorithm to impose both spatial and statistical constraints on attenuation estimation within the MLAA algorithm using Dixon MR images and a constrained Gaussian mixture model (GMM). In this study, we compare the proposed algorithm with MLAA and MLAA_Salomon in brain TOF-PET/MR imaging.

A clinical FDG head PET/CT/MR dataset was used to simulate a 40M-count PET data acquisition with TOF resolution of 580 ps. In MLAA_GMM, Dixon MR images are segmented into outside air, fat/soft tissue classes and an MR low-intensity class corresponding to air cavities, bone and susceptibility artifacts. A mixture of 3 Gaussians (air, fat/soft tissue and bone) was used for the low-intensity class, while uni-modal Gaussians were used for other classes. Bias performance of the algorithms was evaluated against CT-based and 4-class MR-based attenuation correction methods.

Region-of-interest analysis of our simulations showed that the 4-class and MLAA algorithms result in –4.9% and –5.8% bias in soft tissue and –18.5% and –12.4% bias in bone, respectively. Inclusion of MR constrains in MLAA_Salomon and MLAA_GMM resulted in –6.6% and –4.1% bias in soft tissue and –16.1% and –13.0% in bone, respectively. It was found that the performance of MLAA_Salomon depends highly on the robustness of MR segmentation, particularly at air/bone interfaces.

The proposed approach effectively exploits MR prior information and produces attenuation maps that are spatially and statistically more consistent with true attenuation maps.

